# Leveraging particle dynamics in force-fields for network packet routing

**DOI:** 10.1371/journal.pone.0357202

**Published:** 2026-08-27

**Authors:** Subir Biswas, Yida Yang, Amit Kumar Bhuyan, Hrishikesh Dutta, Samir Datta

**Affiliations:** 1 Electrical and Computer Engineering, Michigan State University, East Lansing, Michigan, United States of America; 2 Chennai Mathematical Institute, Chennai, Tamil Nadu, India; Chunghwa Telecom Co. Ltd., TAIWAN

## Abstract

Scalable and resource-efficient packet routing remains a fundamental challenge in modern communication networks, particularly in emerging Internet-of-Things and sensor network deployments where node capabilities are severely constrained. Existing routing paradigms, which includes source routing, hop-by-hop table-driven forwarding and other state-of-the-art frameworks, face inherent scalability limitations arising from either increasing packet header overhead or growing routing table complexity. This paper introduces Particle Dynamics-based Routing (PDR), a novel routing framework inspired by the controllable trajectories of particles in physical force fields. In PDR, network topology is abstracted as a discretized spatial field in which packets are routed along physics-driven trajectories determined by initial launch parameters and pre-assigned logical force distributions at nodes. This enables forwarding decisions at intermediate nodes without reliance on destination-specific routing tables or path-encoded headers. A gravitational-field realization of the framework is presented to demonstrate how routing complexity can be shifted from core network nodes to edge-based pre-launch computation processes. Through theoretical formulation and simulation-based evaluation, the study shows that PDR can maintain constant packet header size independent of network scale, while supporting trajectory-based routing with reduced forwarding overhead at intermediate nodes. The framework further enables natural privacy semantics by limiting exposure of destination information during packet traversal. These characteristics position PDR as a scalable routing alternative for large-scale and resource-constrained network environments. Beyond its immediate applicability, the proposed paradigm establishes a new trajectory-centric perspective on routing design, which opens opportunities for future extensions involving multi-field routing strategies, hierarchical trajectory composition, and routing in higher-dimensional network abstractions.

## 1 Introduction

This article explores an innovative packet routing mechanism inspired by particle movements under force-fields. For instance, the trajectory of a charged particle within an electric field can be controlled by carefully configuring the strength and orientation of the field. Similarly, the trajectory of an object with mass in space can be controlled by applying and configuring a gravitational field distribution [1]. Conversely, for a given gravitational field distribution, the launching velocity (i.e., speed and direction) of an object can be computed to send it from any point to any other point in space. Consider a two-dimensional space in which a gravitational field vector is uniformly applied vertically downwards. Now, from any source point in that space, there exists infinitely possible velocities that can send an object to a specific destination point via projectile trajectories [2]. In other words, it can be said that for a pre-assigned gravitational field distribution, an object can be routed between any two points by launching it with a pre-computable velocity.

Now, let us map this routing approach in a network with planar mesh topology. Visualize the topology as a two-dimensional space, and a logical gravitational field distribution is applied in that space by manually pre-assigning field values and directions at each network node. The objective is to route a packet, which is an analog to the object with mass, from a source network node to a destination network node. The packet is launched with a specific speed and angle from the source node. The concept of speed here is logical, and it is realized by entering its value in the packet header. The launching angle is realized by choosing a specific outgoing link of the source node. Subsequently, a trajectory computation equation is executed at each intermediate downstream node based on the incoming angle (i.e., the incoming link) and the speed information extracted from the packet header. The outcome of that computation at an intermediate node is the corresponding new velocity. This new outgoing speed value replaces the previous speed value written in the packet header, and the packet is forwarded through an outgoing link corresponding the computed angle. This process continues such that the packet traverses through a desired trajectory (i.e., like a projectile) that passes through the intended destination. It is notable that since the notion of speed here is logical, it does not have any bearing on the true speed with which a packet moves through the communication medium. Purpose of the notional incoming speed at a node is to calculate the outgoing angle (i.e., link) and outgoing speed using trajectory computation equations under a logical gravitational field distribution at that node.

This concept of packet routing can extend to particle movement-based routing in other force fields including electrical, magnetic, and electrostatic. Since the routing model works with particle dynamics equations (i.e., relating to particle trajectory in force fields), we term this as Particle Dynamics-based Routing (PDR). It is shown later that the proposed PDR mechanism can shift the majority of routing complexities from a network’s core to its edge. Furthermore, unlike traditional source routing [3] it is achieved without requiring large packet headers which increase with network size. This makes PDR particularly suited for sensor and IoT style networks in which the core sensor nodes are resource-tight in terms of available computing, storage, and energy. Additionally, with PDR, the intermediate nodes in a trajectory do not require access to information on the destination address or MPLS-style label [4] in the packet. This provides a built-in privacy semantics for PDR.

From a broader routing-system perspective, achieving scalable and resource-efficient packet forwarding remains challenging in large and highly constrained network deployments. In particular, routing approaches that rely on per-destination routing tables introduce storage, update, and lookup overhead that grows with network size and dynamics. Conversely, mechanisms that embed route information within packet headers can reduce intermediate-node complexity but lead to header size expansion that limits scalability and energy efficiency in low-power communication environments. These fundamental trade-offs motivate the exploration of alternative routing abstractions that decouple forwarding decisions from both routing-table maintenance and explicit path encoding.

The proposed Particle Dynamics-based Routing (PDR) framework is motivated by the observation that controllable trajectory evolution in physical force fields provides a fundamentally different paradigm for guiding movement through space. By mapping this principle to packet traversal in discretized network topologies, routing decisions can be formulated as local trajectory computations driven by initial launch conditions and pre-assigned logical field distributions. This perspective enables the redistribution of routing complexity toward edge-based trajectory planning while maintaining simple, table-free forwarding operations at intermediate nodes.

## 2 Particle trajectory in continuous field space

In this paper, the concept of PDR is explored with gravity as an example force field. An object’s projectile under gravity in space is initiated at its source point with a launching velocity [2]. Then the object can be thought to be routed by infinite number of intermediate points in space before it reaches the destination. Each intermediate point routes the object based on its current velocity and the point’s local gravitational field vector. This routing-like perspective plays a critical for mapping object projectile into PDR routing.

Discrete-time Trajectory Computation: Consider a situation in which an object’s projectile trajectory needs to be computed under fixed downward gravitational acceleration field vector of magnitude g. If x(t), y(t), s(t),andθ(t) represent the instantaneous location, speed, and the angle respectively with respect to the positive x-axis, the projectile can be expressed using discrete-time difference equations:


$$\begin{array}{c} y(t+\Delta t)=y(t)+s(t)\times \text{sin }(\theta (t))\Delta t \end{array}$$ (1)

$$\begin{array}{c} x(t+\Delta t)=x(t)+s(t)\times \text{cos }(\theta (t))\Delta t \end{array}$$ (2)

The evolution of speed and angle can be captured as:


$$\begin{array}{c} s_{y}(t+\Delta t)=s_{y}(t)-g\times \Delta \text{t} \end{array}$$ (3)

$$\begin{array}{c} s_{x}(t+\Delta t)=s_{x}(t) \end{array}$$ (4)

$$\begin{array}{c} s(t+\Delta t)=\sqrt{s_{x}^{\text{2}}(t+\Delta t)+s_{y}^{\text{2}}(t+\Delta t)} \end{array}$$ (5)

$$\begin{array}{c} \theta (t+\Delta t)={\text{tan}}^{-\text{1}}\frac{y(t+\Delta t)-y(t)}{x(t+\Delta t)-x(t)} \end{array}$$ (6)

Here the space is assumed to be continuous even though the trajectory is computed in discrete time steps Δt. These equations can be solved with an initial condition provided by a launching speed $s_{\text{0}}$ and launching angle $\theta_{\text{0}}$. Eqs. 1–6 are appropriately adapted for PDR routing as follows.

## 3 Particle dynamics-based routing (PDR)

### 3.1 Packet trajectory under discretized field space

**Fig 1:**
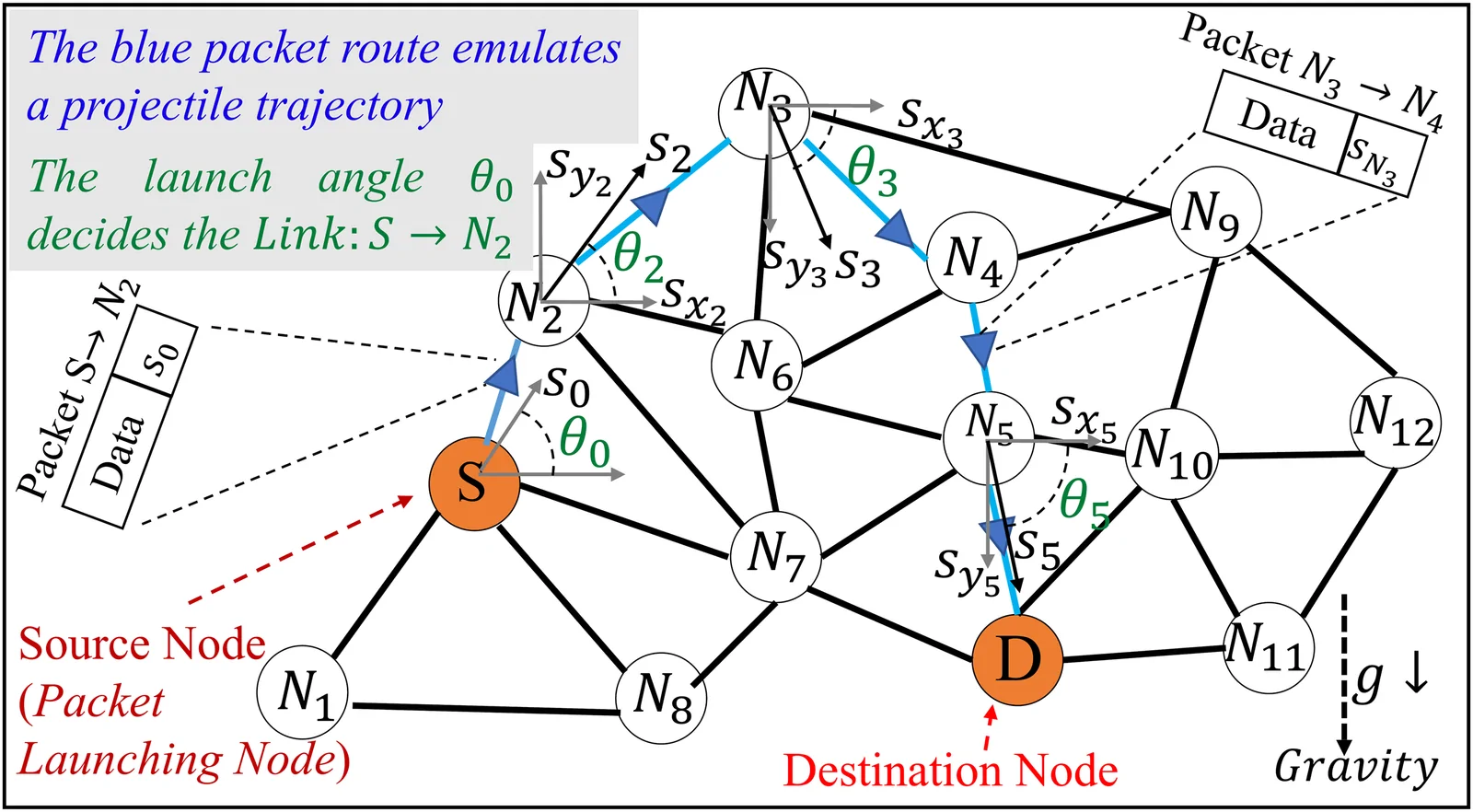
Example gravity-driven PDR routing with discretized space abstraction.


Discrete Field Space: Each node in a network can be considered as a point in a discrete space, which is represented by the network graph. Formally stated, a node is mapped to a point defined by a 2-dimensional cartesian coordinate. Each link of a node possesses a specific angle in that coordinate space. For example, in Fig 1, the link $S-N_{\text{2}}$ has an angle:

$$\begin{array}{c} \theta_{{S-N}_{\text{2}}}={\text{tan}}^{-\text{1}}\left(\frac{y_{S}-y_{N_{\text{2}}}}{x_{S}-x_{N_{\text{2}}}}\right) \end{array}$$ (7)

Gravitational field in such a discrete space is realized by assigning individual field vectors at the network nodes. For example, a downward gravity-triggered projectile packet trajectory can be enabled by assigning the same downward gravity field at all the network nodes. More complex trajectories such as orbital motions can be created by assigning fields at all nodes to be pointed to a single central node in a planar topology.

Packet Trajectory: Unlike in continuous free space, a packet’s trajectory in a network-mapped discrete space is constrained to inter-node links. Moreover, the trajectory of a packet (analogous to a particle) can only change at the nodes, not along the links. An end-to-end route in this context can be seen as a discrete-space approximation of the continuous free-space trajectory described in Equations 1–6. As the network graph becomes denser, the degree of space discretization decreases, resulting in routes that more closely resemble free-space projectiles.

PDR Routing Overview: Routing of a packet follows a trajectory decided by its launching speed and angle (i.e., $s_{\text{0}}$ and $\theta_{\text{0}})$at the source node (i.e., NodeS in Fig 1). The source node forwards the packet through one of its links that corresponds to the launching angle $\theta_{\text{0}}$ most accurately. It also writes the speed $s_{\text{0}}$ in the packet header before forwarding. For example, the source NodeS in Fig 1 writes the speed $s_{\text{0}}$ in the packet header and forwards it to the next hop $NodeN_{\text{2}}$.$NodeN_{\text{2}}$ is selected because among all the outgoing links of NodeS, the $Link:S\to N_{\text{2}}$ corresponds to the launching angle $\theta_{\text{0}}$ most accurately.

Upon receiving the packet, $NodeN_{\text{2}}$ extracts the incoming speed value from the packet header, and it infers the incoming angle based on the incoming link (i.e., Link: $S\to N_{\text{2}}$). Using this incoming speed and angle information, its one-hop topological information, and the preassigned local gravitational field vector, intermediate node $N_{\text{2}}$ computes the packet’s outgoing speed and angle. As shown in Fig 1, the outgoing speed computed at $NodeN_{\text{2}}$ is $s_{\text{2}}$, and the Link: $N_{\text{2}}\to N_{\text{3}}$ corresponds to the outgoing angle $\theta_{\text{2}}$ most accurately. After deciding the next hop (i.e., $N_{\text{3}}$), $NodeN_{\text{2}}$ replaces the speed value in the packet header by the newly computed outgoing speed $s_{\text{2}}$, and forwards it to $NodeN_{\text{3}}$. This process continues till the packet reaches its destination.

It should be noted that in Fig 1 and the following figures, the packet’s destination address is not shown in the packet header. This is to highlight the fact that a packet’s destination is not used for packet forwarding at the intermediate nodes. In reality, an encrypted version of the destination address is included so that the actual destination can identify a packet that is meant for itself. More about this will be presented in Section 7.

## 4 Packet launch and route computation

A packet is launched at its source node with a velocity represented by speed and angle $s_{\text{0}}$ and $\theta_{\text{0}}$ respectively. This velocity is determined by a route search process (see Section 10) such that for the given network wide gravitational field distribution, the resulting route/trajectory is guaranteed to pass through the intended destination node. The source node S writes the quantity $s_{\text{0}}$ in the packet header before forwarding it via the link that corresponds to the angle $\theta_{\text{0}}$ most accurately.

### 4.1 Route computation

The route/trajectory computation starts at the first downstream node (i.e., $N_{\text{2}}$ in Fig 1) based on its locally assigned gravitational field vector, the packet’s speed value retrieved from its header, and the arrival angle interpreted from the link through which the packet is received. The continuous-space trajectory computation equations from Section 2 are adapted by node $N_{\text{2}}$ for trajectory computation in the discretized space as explained in Section 3.

Upon receiving the packet, the node $N_{\text{2}}$ at coordinates ($x_{N_{\text{2}}},y_{N_{\text{2}}}$) finds its next hop using the following equations in discrete time steps Δt.


$$\begin{array}{c} x_{p}=x_{N_{\text{2}}}+s_{\text{0}}^{x}\times \Delta t \end{array}$$ (8)

$$\begin{array}{c} y_{p}=y_{N_{\text{2}}}+s_{\text{0}}^{y}\times \Delta t \end{array}$$ (9)

The terms $s_{\text{0}}^{x}$ and $s_{\text{0}}^{y}$ are the discrete analogue of vertical and horizontal components of speed in the continuous space. And Δt is the time-step for which the next point in a packet’s trajectory is computed. Eqns. 8 and 9 produce a notional point p in continuous space where the packet should be routed next. However, given that the space is discrete, point p may or may not correspond to a real network node, which are the only feasible points in this discrete space. If a node corresponding to p’s coordinates does not exit, node $N_{\text{2}}$ does an approximation by choosing a node $N_{\text{3}}$ as the next hop such that the angle of $Link:N_{\text{2}}\to N_{\text{3}}$ is closest (i.e., out of all links of $NodeN_{\text{2}}$) to the angle $\theta_{N_{\text{2}}-p}$, which is defined as:


$$\begin{array}{c} \theta_{N_{\text{2}}-p}={\text{tan}}^{-\text{1}}\left(\frac{y_{p}-y_{N_{\text{2}}}}{x_{p}-x_{N_{\text{2}}}}\right) \end{array}$$ (10)

Here $\theta_{N_{\text{2}}-p}$ is the angle made by the straight line connecting the PDR-computed point p and node $N_{\text{2}}$. Formally, the choice of the next link to be traversed by the packet is dictated by the following condition:


$$\begin{array}{c} Link:N_{\text{2}}-o=\text{min }(|\theta_{N_{\text{2}}-p}-\theta_{N_{\text{2}}-o}|)\text{\textasciitilde{}for\textasciitilde{}any\textasciitilde{}}o\in {𝒪}_{N_{\text{2}}} \end{array}$$ (11)

Here, the term$O_{N_{\text{2}}}$ is the set of all one-hop neighbors [5] of $N_{\text{2}}$. The term$\left|\theta_{N_{\text{2}}-p}-\theta_{N_{\text{2}}-o}\right|$ corresponds to the absolute difference between an actual link angle $\theta_{N_{\text{2}}-o}$ and the angle made by the PDR-computed point p with $NodeN_{\text{2}}$.

**Fig 2:**
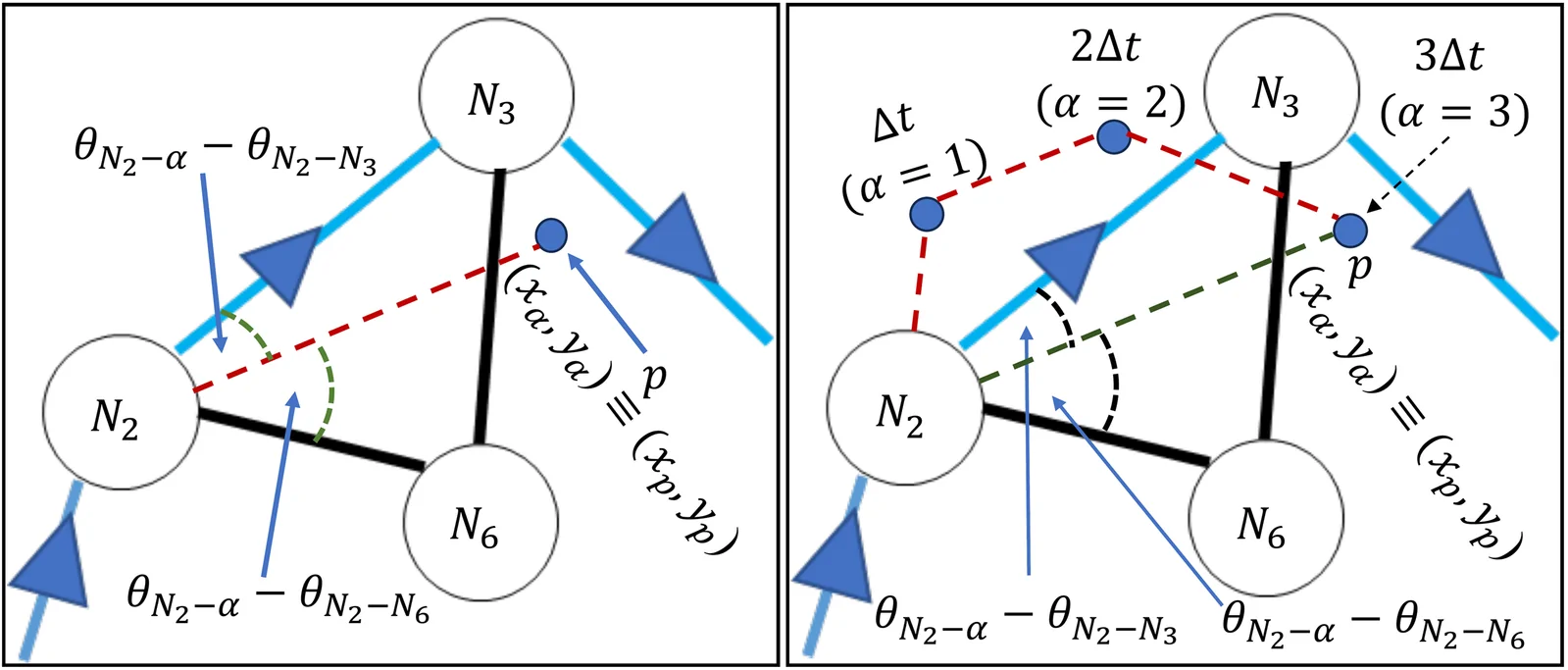
(left) Example of approximated next-hop selection in PDR; (right) Iterative approximation of next-hop to address discretized space.

The condition in Eqn. 11 is depicted via the example in Fig 2. It shows that the angle corresponding to the $Link:N_{\text{2}}\to N_{\text{3}}$ link is closest with the computed outgoing angle $\theta_{N_{\text{2}}-p}$, which leads $NodeN_{\text{2}}$ to choose $NodeN_{\text{3}}$as the next-hop node.

The mechanism described so far ensures that the packet is forwarded in the right direction through the discrete topological space in a piece-wise continuous manner. One important question regarding this strategy: Is the outgoing speed of a packet sufficient for it to reach the next-hop node in time Δt? The question arises because: i) the next hop-node (i.e., ${NodeN}_{\text{3}}$ in Figs 1 and 2) is an approximation of the computed true next-hop point p, and ii) the physical length of the link to the next-hop (i.e., $Link:N_{\text{2}}\to N_{\text{3}}$) has no bearing on the computation of the point p. To mitigate these, reachability to the next hop node is ensured by computing point p iteratively using Eqns. 8 and 9 till it corresponds to a next hop node (i.e., $N_{\text{3}}$) that is physically reachable. Such iterations are executed as:


$$\begin{array}{c} x_{\alpha }=\left\{ \begin{array}{c} @lx_{\alpha -\text{1}}+s_{\text{0}}^{x}\times (\alpha -\text{1})\Delta t,\;for\alpha >\text{1}\\ x_{N_{\text{2}}},\;for\alpha =\text{1} \end{array}\right. \end{array}$$ (12)

$$\begin{array}{c} y_{\alpha }=\left\{ \begin{array}{c} @ly_{\alpha -\text{1}}+s_{\text{0}}^{y}\times (\alpha -\text{1})\Delta t,\;for\alpha >\text{1}\\ y_{N_{\text{2}}},\;for\alpha =\text{1} \end{array}\right. \end{array}$$ (13)

These are executed at the current node (e.g., $N_{\text{2}}$) till the following condition is met:


$$\begin{array}{c} ||\left(x_{\alpha },y_{\alpha }\right)-(x_{o},y_{o})||<||\left(x_{\alpha },y_{\alpha }\right)-(x_{N_{\text{2}}},y_{N_{\text{2}}})||\text{\textasciitilde{}for\textasciitilde{}any\textasciitilde{}}o\in {𝒪}_{N_{\text{2}}} \end{array}$$ (14)

The term $O_{N_{\text{2}}}$ is the set of all one-hop neighbors of $N_{\text{2}}$, and the iteration parameter α has the following significance. Within a time-step Δt, the point ($x_{\alpha },y_{\alpha }$) may not satisfy the condition stated in Eqn. 17. In other words, the time duration Δt may not be sufficient for a packet to reach from $NodeN_{\text{2}}$ to a computed point p, which can be approximated to a real one-hop neighbor node of $N_{\text{2}}$. If not, $N_{\text{2}}$ iteratively computes for another duration Δt to see if the next computed point p can approximate a next-hop node that is physically reachable after that new Δt. This iterative process is continued till a physically reachable next-hop neighbor of $N_{\text{2}}$ can be found corresponding to a computed point p. This iterative process is explained in Fig 2 which shows the intermediate PDR-computed points (i.e., at $x_{\alpha },y_{\alpha }$) till a point p with coordinates $\left(x_{p},y_{p}\right)$ is achieved with the condition stated in Eqn. 17 is satisfied.

Once the next hop neighbor (i.e., $N_{\text{3}}$) is decided, the packet’s new speed before it leaves the current node (i.e., $N_{\text{2}}$) is computed as:


$$\begin{array}{c} s_{N_{\text{2}}}^{x}=s_{\text{0}}^{x} \end{array}$$ (15)

$$\begin{array}{c} s_{N_{\text{2}}}^{y}=s_{\text{0}}^{y}-g\Delta t \end{array}$$ (16)

$$\begin{array}{c} s_{N_{\text{2}}}=\sqrt{{\left(s_{N_{\text{2}}}^{x}\right)}^{\text{2}}+{\left(s_{N_{\text{2}}}^{y}\right)}^{\text{2}}} \end{array}$$ (17)

Node $N_{\text{2}}$ puts the computed speed $s_{N_{\text{2}}}$in the packet header before forwarding it to the next hop $NodeN_{\text{3}}$. Upon reception of the packet, $N_{\text{3}}$executes the steps described in Eqns. 7–14 in order to find the next hop. This process continues till the packet reaches its destination. The full PDR logic is represented in the following Algorithm-I.

Algorithm I: Details of the PDR algorithm logic

**1: Given:** Network topology, Node coordinates, Gravity information, Acceleration of gravity g,Gravity direction $\vec{g}$**,** Termination threshold ($s_{th},\theta_{th}$)

**2: Input:** Source node (S), Destination node: D, Initial speed: $\left(s_{\text{0}}^{x},s_{\text{0}}^{y}\right),$Initial launching angle: $\theta_{\text{0}}$

**3: Variables:** $N_{curr}$ ( Current node), $N_{next}$ (Next node that packet is going to), $D_{a-b}$ (Distance between two points/nodes a and b), $\theta_{a-b}$ (Angle between two points/nodes *a* and *b* w.r.t cartesian x-axis)

**4: Algorithm:**

**5: IF** $N_{curr}$ is S: // The next node $N_{next}$is known, which is the node where the node corresponds to $\theta_{\text{0}}$

**6:** // Calculate the scalar of Δt to $N_{next}$

**7:**  **IF** $s_{\text{0}}==\text{0}:$

**8:**    $\alpha =\frac{D_{\text{0}-next}}{\Delta t}$    // Refer Eqns. 8–11

**9:**  ELSE:

**10:**   $\alpha =\frac{(\frac{D_{0-next}}{S_{0}})}{\Delta t}$

**11:**  **END IF**

**12:** // Calculate $s_{next}and\theta_{next}$when packet arrives at next node

**13:** // All calculation is done at the node, and $s_{next},\theta_{next}$ are all the data packet is carrying.

**14:** $s_{next}^{x}=s_{\text{0}}^{x}$

**15:** $s_{next}^{y}=s_{\text{0}}^{y}-g*\alpha *\Delta t$

**16:** $\theta_{next}=\theta_{o}$ **IF** $N_{curr}$ is S **ELSE** $\theta_{next}=\theta_{curr}$ **END IF**

**17: ELSE:**

**18:** // Calculate an abstract point p

**19:** $x_{p}=x_{curr}+s_{curr}^{x}*\alpha *\Delta t$

**20:** $y_{p}=y_{curr}+s_{curr}^{y}*\alpha *\Delta t$

**21:**  **IF** $\left(x_{p},y_{p}\right)$ is same coordinate as one of the neighbors of $N_{curr}$:

**22:  /**/ The next node is found, next node is the node corresponds to p.

**23:**  $N_{next}=N_{p}$

**24:** **ELSE:**

**25:** $\theta_{curr-p}={\text{tan}}^{-\text{1}}\left(\frac{y_{p}-y_{curr}}{x_{p}-x_{curr}}\right)$

**26:**   // Find the next possible node $N_{np}$

**27:**   $N_{np}={\text{argmin}}_{\forall O_{curr}}\left(\left|\theta_{curr-p}-\theta_{curr-neighbors}\right|\right)$

**28:**   **IF** $D_{curr-p}\geq D_{curr-np}$**:**

**29:**    // The next node is found

**30:**   $N_{next}=N_{np}$

**31:**   $\theta_{curr}=\theta_{curr-next}$

**32:**  **ELSE:**

**33:**  α++

**34:**   Goto Step 14

**35:**  **END IF**

**36:** **END IF**

**37:** // Once the $N_{next}$ is found, check the stopping criteria

**38:** **IF** $N_{next}==D:$

**39:**   Forward the packet to $N_{next}$

**40:**   Terminate    //Packet reaches destination

**41:** **ELSE:**

**42:   IF** $y_{next}\leq s_{th}$ && $\left|\theta_{next}-\vec{g}\right|\geq \theta_{th}$:

**43:**    Terminate   //This is the end of the trajectory

**44:**   **ELSE:**

**45:**    Goto Step 14.

**46:**  **END IF**

**47:** **END IF**

**48: END IF**

Note that the algorithm works without any changes near the topology boundaries. When a packet reaches the border, and the next PDR-computed coordinate is outside the border, the algorithm selects the next node in the topology based on the usual closest-angle approximation towards that outside point. Fig 6 demonstrates the resulting packet trajectories in such near-border situations.

### 4.2 Pre-launch route search

The PDR mechanism above details route computation at the intermediate nodes of a trajectory. It does not address the process of determining the packet launching velocity at the source node. The launching velocity is determined using a pre-launch route search process. Such a route-search entity, which can be the source node itself or can be a separate outside server, needs access to the network topology information. The search process can be fully algorithmic, heuristics based, or brute force exploration depending on the size of the network and the resource availability for such search. Using a brute force exploration, for example, the route-search entity would explore all possible trajectories emanating from the source node with; i) a given gravitational field distribution across the network, ii) launching angles corresponding to all outgoing links of the source node, and iii) a set of launching speeds. One feasible trajectory from all those explored will be chosen, and the corresponding launching speed and angle will be used by the source node. Specific route search mechanisms for our experimental results will be presented in Section 10.

From a computational perspective, the pre-launch route search process introduces an edge-centric complexity that depends on the size of the trajectory search space, which is influenced by factors such as network topology density, the granularity of candidate launching speeds and angles, and the number of field directions considered in multi-field extensions. However, this computational cost is incurred prior to packet transmission and does not affect per-hop forwarding latency during packet traversal. Moreover, since a computed trajectory can be reused for multiple packets with identical or similar source-destination attributes, the effective amortized cost per packet can remain low in steady traffic conditions. This separation between route computation overhead and forwarding simplicity represents a key design trade-off in PDR which enables reduced processing burden at intermediate nodes while maintaining flexibility in route determination.

From an operational standpoint, the trajectory computation mechanism in PDR also enables adaptation to dynamic network conditions such as node failures, mobility-induced topology variations, or intermittent connectivity disruptions. Since packet routes are not stored as persistent state within intermediate nodes, topology changes do not necessitate routing-table updates across the network. Instead, when previously computed trajectories become infeasible due to altered connectivity conditions, updated launch parameters can be obtained through renewed trajectory search procedures. This edge-centric re-planning capability allows PDR to maintain routing feasibility under evolving network conditions while preserving lightweight forwarding operations during packet traversal.

### 4.3 Non-gravitational force fields

PDR can be realized by leveraging particle dynamics under various other force fields including electrostatic, electromagnetic etc. With electrostatic, packets would act as charged particles whose trajectories will be dictated by discretized versions of Coulomb’s law [6]. In contrast to gravity, in this case, the polarity of a particle with respect to field distributions will provide additional degrees of freedom, and therefore finer grain of control for packet routing. With electromagnetism, the trajectory of a packet, which is the analog of a charged particle, will be determined using a set of discretized versions of Maxwell’s equations [7].

## 5 Related prior work

This section reviews existing relevant routing mechanisms and contrasts them with the propose paradigm. Protocols in source routing family determine the complete path for a packet at the source node and embed the path information in the packet header. The primary advantage is that intermediate nodes can forward packets without complex computations. However, the worst-case packet header size grows linearly [8] with network diameter, leading to serious scalability concerns. Hybrid source routing [9] has been attempted to address this in which deterministic routes are combined with probabilistic adjustments to adapt to network dynamics. While mitigating the packet header scalability to some extent, this approach introduces additional computational complexity at the source, and challenges related to stale information in dynamic environments [8]. Dynamic source routing schemes that incorporate path compression methods [9] have also been proposed for making source routing more scalable. These methods reduce header size but introduce complexities in packet parsing and forwarding. By deploying a novel trajectory-based routing, the proposed PDR paradigm in this paper addresses the packet header scalability issues inherent in the protocols in source routing family.

Hop-by-hop routing based on algorithms such as Bellman-Ford and Dijkstra, the source and all the downstream intermediate nodes forward packets based on per-destination routing tables. These tables are pre-computed and periodically updated based on network topology and traffic conditions. Most of the traditional IP protocols including RIP, OSPF, and BGP [10] rely on this principle. Unlike in source-routing, the route computation and the routing table storage burdens are placed in the intermediate nodes in the network core. As a tradeoff, the overhead of carrying the list of intermediate nodes, as done in source routing, is avoided here. The scalability issue here comes from the complexities of per-node path computation and routing table storage, both of which grows linearly or faster with the network size.

More recent approaches such as Software Defined Network (SDN) [11] address the scalability by distributing and reconfiguring routing loads across different parts of a network. The overhead and scalability in these approaches are shifted to the operations of periodic routing table exchanges and control connections across route-servers and network nodes.

Specialized applications of hop-by-hop routing have been observed in vehicular networks [12] and IoT ecosystems [13]. While incorporating many application- and network-specific optimizations, their core hop-by-hop approach still suffers from algorithmic scalabilities outlined above.

Named Data Networking (NDN) [14], which forwards content based on content name/identity instead of IP addresses. While NDN can improve retrieval latency and network bandwidth load by smart caching of contents physically close to consumers [15], it still relies on content routing table formation and maintenance in network nodes. As a result, the core scalability issues of hop-by-hop routing, as pointed above, are still present for NDN.

There is a large body of literature on mobile hop-by-hop routing that are centered around updating routing tables in order to cope with time-varying network graphs. Such protocols include mobile ad hoc networks (MANET) routing [16] such as *ad hoc* on-demand distance vector (AODV) [17], Destination-Sequenced Distance Vector (DSDV) [18], and Dynamic Source Routing (DSR) [19]; Opportunistic Routing (OR) [20] such as Link State-aware OR [19], Geographic OR [21], and Code-aware OR [22]; Delay Tolerant Network (DTN) routing [23] such as Bundle-based forwarding (BF) [24], Queued forwarding (QF) [25], and Opportunistic forwarding (OF) [26]. While making specific accommodations for mobility, all these protocols deploys some form of hop-by-hop forwarding, thus inheriting its core scalability shortcomings (Table 1).

**Table 1:** Comparison between PDR and classical routing techniques.

Mechanism	Packet Header Size	Location of Route Computa-tion	Routing Table Size and Scaling	Routing Table Formation Complexity	Packet Forwarding at Intermediate Nodes	Generalized Min-hop Routing	Reachability	Built-in Privacy
**Source-Routing**	Scales with route length	Edge	Table not Needed	Not needed	Not needed	Feasible	Complete	None
**Hop-by-hop Routing**	Constant	Core	Scales with network size	Scales with network size	Scales with routing table size	Feasible	Complete	None
**Label Switching (MPLS)**	Constant	Core	Scales with # of flows through node	Scales with network size	Scales with routing table size	Feasible	Complete	None
**Particle Dynamics- based Routing (PDR)**	Constant	Edge / Route Sever	Table not needed	Not needed	Complexity of PDR computation (Constant)	Not feasible	Partial (See Section 7)	Yes

Many of these existing protocols introduce routing hierarchies at different scales in order to ease the scalability issues, but the core issues in hop-by-hop routing still persist in some form. The proposed PDR mechanism in this paper avoids the fundamental shortcoming of hop-by-hop routing by replacing table lookups with field-based trajectory computations. This can achieve scalability while not having to maintain large packet headers as needed for source routing.

In Asynchronous Transmission Mode (ATM) [27,28] and more recent label-based virtual circuit-oriented approaches such as MPLS [4], the packet forwarding complexity is reduced by replacing full-blown routing-table searches with smaller label-table searches. The size of a routing table in hop-by-hop packet forwarding scales with the networks size. In contrast, the size of a label table in MPLS scales with the number of MPLS/Virtual-circuit (VC) flows passing through a node, which is usually much smaller than the number of network nodes. As a result, the label table search complexity can be significantly lower than that for routing table search in hop-by-hop routing. The packet size in MPLS is also reduced by replacing the destination node address by an MPLS/VC flow label. While achieving these improvements, the MPLS/VC label table formation still incurs storage and computational burdens, which is borne by the core network nodes. While various levels of Quality-of-Service provisioning, routing hierarchy, and combinations of the classical routing mechanisms can be implemented for better scalability, the above observations about packet header length and computational and storage complexities generally remain applicable to those optimized methods. The proposed PDR mechanism in this paper addresses MPLS’ storage and computational burdens.

Geographical routing [29] such as Greedy Perimeter Stateless Routing (GPSR) utilizes nodes’ absolute location information to make packet forwarding along computed trajectories. decisions. This approach is particularly advantageous in static sensor networks with *a priori* known node coordinates. One major shortcoming of geographical routing is its inability to handle large voids in a network topology where trajectories can prematurely terminate during packet forwarding. The proposed PDR approach has similarities with geographical routing in that it also perform trajectory based forwarding. However, it does so using the concept of virtual force-fields instead of geographical information, which can be limiting in many application scenarios.

Optimized flooding based mechanisms such as Ant colony optimization (ACO) routing (e.g., AntNet [30]) gradually prune packet flooding overhead over time using virtual pheromone concentrations. These protocols adaptively discover routes by probabilistically selecting paths based on pheromone concentrations [31]. Primary scalability for these protocols come from the fact that they rely on continuous traffic flow without which pheromone concentrations cannot be practically realized. The proposed PDR method does not rely on such constancy of packet flows and provides scalable routing.

## 6 Qualitative distinctions of PDR

### 6.1 PDR advantages

First, a fundamental distinction between Particle Dynamics-based Routing (PDR) and conventional source-routing mechanisms lies in how end-to-end paths are represented and enforced during packet traversal. In traditional source routing, the source node explicitly encodes the sequence of intermediate nodes or links in the packet header, thereby requiring intermediate nodes to forward packets according to pre-specified path information. In contrast, PDR determines packet traversal implicitly through trajectory evolution governed by the packet’s launching parameters and pre-assigned logical force-field distributions at network nodes. As a result, intermediate nodes execute local trajectory computations rather than parsing or interpreting path-specific header information. This enables route realization without packet header growth proportional to path length, while still allowing route determination to be performed prior to packet launch.

Second, maintaining a constant packet header size in PDR provides several practical advantages in large-scale and resource-constrained networks. Since header length does not grow with route length or network diameter, communication overhead remains predictable and bounded, which is particularly beneficial in low-bandwidth wireless environments. This property also contributes to improved energy efficiency at transmitting nodes by limiting per-packet transmission cost. From a processing standpoint, constant header size simplifies packet parsing and forwarding operations at intermediate nodes that enables lightweight implementation in devices with limited memory and computational capability. Furthermore, by decoupling routing scalability from packet format expansion, PDR allows network growth without requiring proportional increases in protocol overhead, thereby supporting more stable performance under increasing network size and density. The packet headers in PDR (e.g., more generally in mPDR) need to carry only two control fields, namely, the speed, and the force field vector direction. The size of these header fields are constant, and independent of the route length or network size. This is a distinct advantage over source routing which requires packets with header length that increases with the network size.

Third, a key scalability advantage of PDR arises from its routing-table-free operation at intermediate nodes. In conventional routing protocols, such as hop-by-hop routing, nodes must maintain per-destination state information that grows with network size and requires periodic updates to reflect topology changes. This introduces storage overhead, control traffic for routing-table dissemination, and additional lookup latency during packet forwarding. In contrast, PDR eliminates the need for such routing tables by enabling forwarding decisions through local trajectory computations based on packet motion parameters and pre-assigned logical field values. As a result, intermediate-node state complexity remains largely independent of network scale that allows the network to grow in size or density without proportional increases in memory requirements or routing maintenance overhead. This characteristic supports improved scalability, particularly in dynamic or resource-constrained environments where minimizing per-node control burden is critical. The absence of routing tables in PDR makes it more scalable than MPLS/Virtual-circuit style routings as well.

Finally, due to the fact that no explicit destination-specific information is carried within the packets, a snooping agent in an intermediate node can infer very little about a packet’s destination. Using the speed information and the incoming link (i.e., angle), an intermediate snooping node can potentially infer the remaining part of the packet’s trajectory if the global topology information is available to that node. While this trajectory information can be inferred, the exact identity of the specific destination still remains hidden. This provides a certain amount of natural privacy in PDR.

Discussion: From a broader routing architecture perspective, PDR exhibits distinct scalability characteristics when compared with dynamic routing paradigms such as Software Defined Networking (SDN) and Named Data Networking (NDN). In SDN-based approaches, routing intelligence is often centralized within controllers that maintain global topology awareness and install forwarding rules at network nodes, which can introduce control-plane overhead and scalability challenges in highly dynamic or large-scale deployments. Similarly, NDN architectures rely on stateful forwarding mechanisms involving Pending Interest Tables and content caching structures that grow with traffic demand and network size. In contrast, PDR enables packet forwarding through trajectory evolution governed by local computation equations without requiring persistent routing or request state at intermediate nodes. This trajectory-centric design allows routing complexity to be concentrated at the route planning stage while maintaining lightweight forwarding operations, thereby offering an alternative scalability pathway for resource-constrained and large-scale network environments.

### 6.2 PDR shortcomings

While reducing the burden in the network core nodes, a pre-launch route search process as explained in Section 10 needs to be executed. Such a search entity, either the source node or a separate route compute server, needs to have access to the full network topology information. Even though one such search can serve all packets with the same source-destination attributes, like in source routing, this does add complexity at the edge of the network. To be noted, that once a packet is launched after that route search, all the PDR’s advantages mentioned above hold at the network core nodes.

The other disadvantage stems from the non-minimum-hop nature of the PDR trajectories. With gravity-driven PDR, as described in this article, all routes are discrete approximations of continuous-space projectiles. Similar trajectory eccentricities are expected to be present for the non-gravity force fields as well. As presented in Section 8, in certain special situations, those non-minimum-hop PDR trajectories can be leveraged for multicast routing to multicast group member nodes that are located on a PDR route trajectory.

Finally, because PDR works with a graph modeled as discretized space, sparse topologies can give rise to route infeasibility issues. A detailed characterization of such infeasibility and approaches to mitigate that will be presented in Sections 7 and 8.

To summarize, PDR can shift the majority of routing complexities from network core to the edge. It is achieved while avoiding the key limitation of source routing, namely, long packet header size that scales with network size. This makes PDR particularly attractive for low-power embedded network nodes with extreme energy constraints, and limited communication bandwidth that favors small constant size packet headers. Additionally, in the absence of destination addresses or MPLS-style labels [4] in the packet headers, very little information about the destination is exposed to adversarial agents in intermediate nodes. This provides a built-in privacy semantics for PDR. Table I summarizes the comparison between PDR and the classical routing approaches.

## 7 Reachability and multi-field PDR

In a continuous force field, there exists infinite number of possible particle trajectories between any two points. In other words, the reachability from any source to any destination is guaranteed. Due to the space discretization in PDR, however, such a guarantee is absent. The sparser the network, the discreteness in space becomes more pronounced, thus leading to higher likelihood of unreachability.

**Fig 3:**
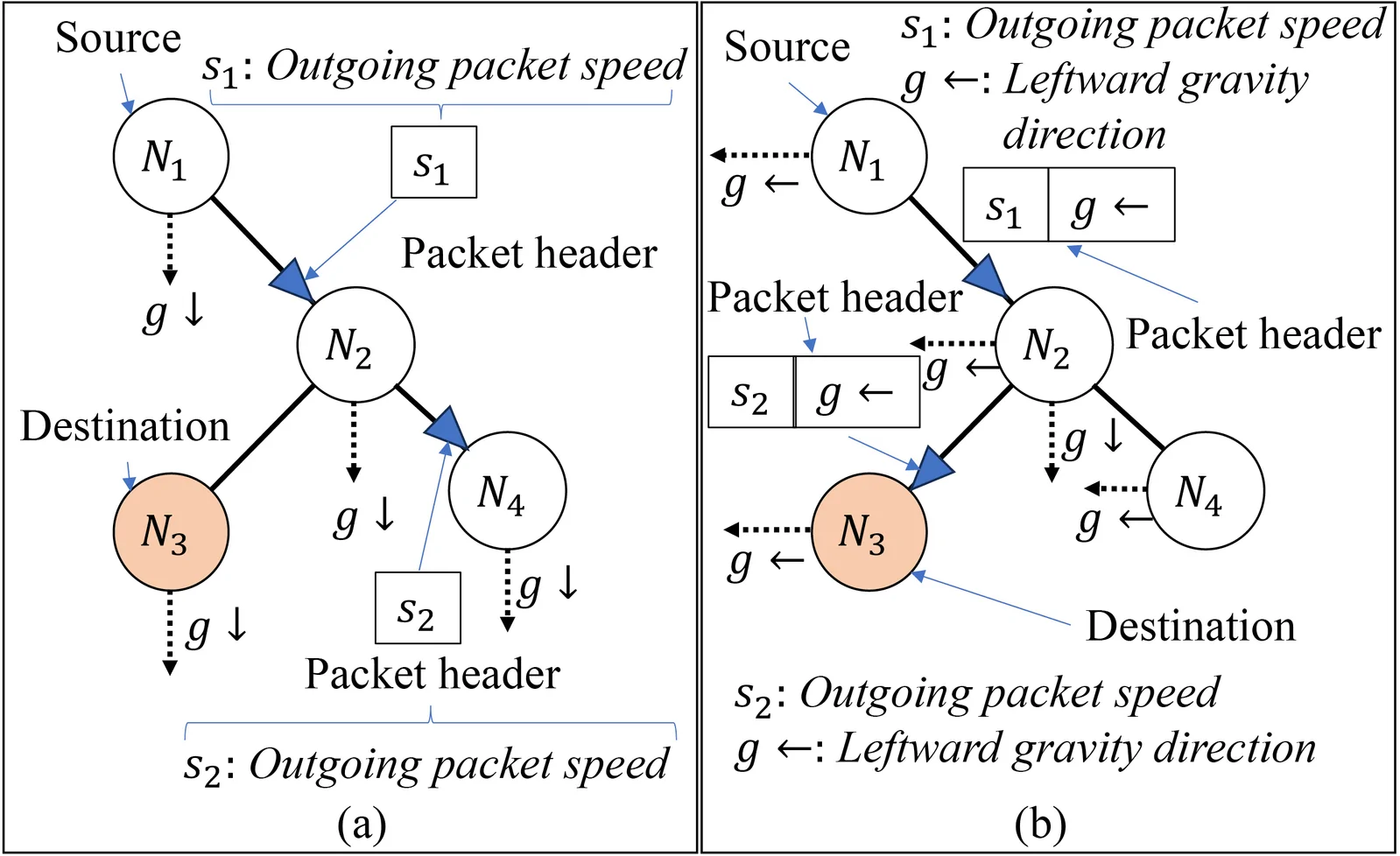
(a) Unreachability with PDR, (b) Enhancement with Multi-field PDR.

One such example is shown in Fig 3a, where a desired trajectory $N_{\text{1}}\to N_{\text{2}}\to N_{\text{3}}$ does not exist based on the baseline PDR presented so far. Based on the network wide downwards gravitational field, node $N_{\text{1}}$ launches a packet to $N_{\text{2}}$ as the next hop towards destination $N_{\text{3}}$. The diagram shows the content of packet header that includes the outgoing speed $s_{\text{1}}$. Upon reception of the packet, based on its local pre-assigned gravitational field vector, node $N_{\text{2}}$ executes PDR. The algorithm points to node $N_{\text{4}}$ instead of $N_{\text{3}}$ as the next hop. Hence, the route $N_{\text{1}}\to N_{\text{2}}\to N_{\text{3}}$ is not feasible with the specific pre-assigned filed values and the launching speed and angle at $NodeN_{\text{1}}$. This lack of reachability for a source-destination pair can be absolute when for any possible network-wide field direction, there exists no packet launching speed and angle at $NodeN_{\text{1}}$ such that the packet’s trajectory traverses through $NodeN_{\text{3}}$.

Multi-field PDR (mPDR): Reachability issues can be significantly mitigated by an augmentation referred to as multi-field PDR. With mPDR, the route search process explores PDR trajectories with many different field directions at all nodes in the network. For instance, assuming that the network wide gravitational field vector can be in one of 8 directions, the search process evaluates PDR trajectories for each of those eight field directions to find a feasible route. Multiple field directions expand the size of the searched route pool, thus improving end-to-end reachability. A detailed performance analysis of reachability with and without mPDR is presented in the results Section 10.

The use of multiple gravitational vectors in mPDR effectively enlarges the trajectory design space available during the pre-launch route search process. Instead of constraining packet motion to trajectories governed by a single global field orientation, the route search entity evaluates potential packet paths under several candidate field directions. Each field orientation induces a different set of trajectory curvatures and directional tendencies in the discretized topology, thereby enabling the discovery of routes that may not be feasible under a single-field configuration. By selecting the field direction that yields a valid or more favorable trajectory toward the destination, mPDR improves routing feasibility and provides additional flexibility in shaping packet traversal patterns, particularly in networks with irregular connectivity or spatial sparsity.

It is important to note that trajectory realization in mPDR is not explicitly designed to minimize classical shortest-path metrics such as hop count or geometric distance. Instead, the routing process prioritizes trajectory feasibility under logical field constraints, which may lead to paths that differ from conventional shortest-path solutions.

Fig 3b shows the impacts of mPDR to achieve reachability via exploring two different field directions. While the route search fails for downwards gravity, a feasible route can be found with gravity pointing to the left. This gravity direction is entered in the packet header before launching such that all intermediate nodes can use this left-pointing gravity for executing the mPDR algorithm. In other words, once the gravity direction is decided by the route search process, all intermediate nodes use that direction (i.e., available in the packet header) for packet forwarding using Eqs. 8-17.

With mPDR, the network wide reachability increases with the number of distinct field directions that are considered during the pre-launch route search. For example, an eight-direction mPDR would have better reachability compared to the baseline PDR, which is a degenerate case of mPDR with only one field direction. Experimental results in Figs 9 and 10 demonstrate that complete or near-complete reachability can be achieved by allowing mPDR routing to use a large number of diverse field directions.

It is notable that with higher number of field directions, there is no increase in the computational complexity of mPDR at the intermediate nodes. This is because they still execute mPDR with only one field direction that was received in the packet header. The computation overhead of the pre-launch route search process, however, linearly increases with the number of field directions.

A natural question at this stage is how to determine the filed magnitude. It turns out that after a field direction is adopted, the effects of field magnitude in PDR/mPDR can be captured by correspondingly choosing an appropriate launching speed at the source. Therefore, a reasonable strategy is to keep the field magnitude fixed and vary the launch speed for exploring and finding feasible end-to-end routes during the pre-launch route search process.

mPDR with Hierarchical Source Routing (mPDR-HS): There could be scenarios, especially for very sparsely connected topologies, in which mPDR with many field directions can still fail to discover feasible routes for specific source-destination pairs. Creating a hierarchically source routed version of mPDR can mitigate those scenarios. With mPDR-HS, the route search process attempts to find a number of route segments such that: i) they are individually feasible (i.e., reachable) using mPDR, and ii) when concatenated, they realize a valid end-to-end route. Each of those segments have its own {launching speed, angle, and a gravitational field direction}. This tuple for each segment is inserted in the packet header before launching it to the corresponding segment. This is similar to hierarchical source routing in that the segments need to be identified before launching, and a list of the end-points of the segments needs to be put in the packet header. This makes the packet header longer than the baseline PDR/mPDR.

**Fig 4:**
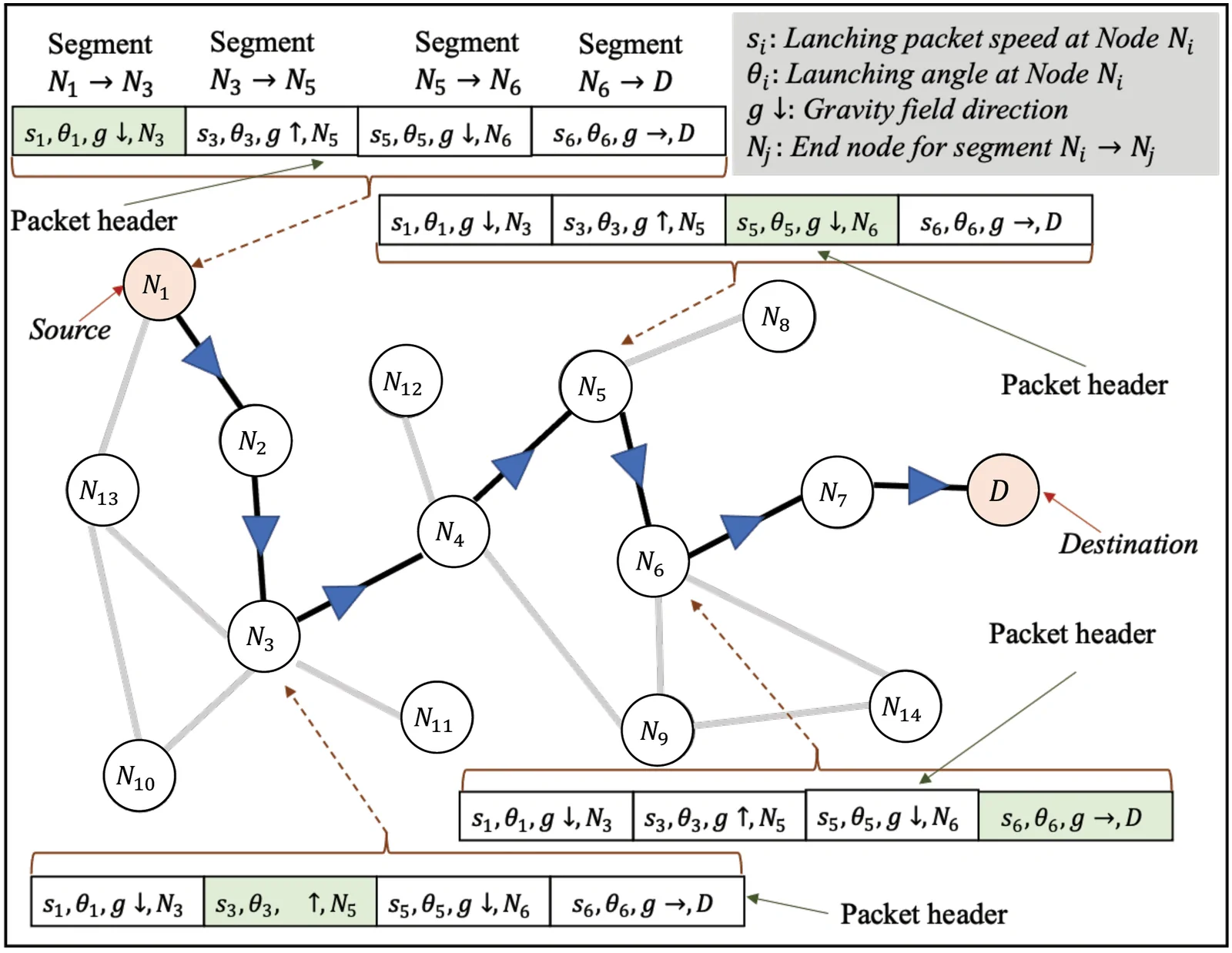
*mPDR* with Hierarchical Source Routing (*mPDR-HS*); the launching speed, angle, field direction, and segment end point corresponding to the relevant segment are highlighted in green.

The scenario is explained by an example in Fig 4. In this topology, using baseline PDR with downward gravitational field, no route/trajectory can be found from $N_{\text{1}}$ to D. Using mPDR with four field directions does not produce a feasible route either. As shown, this can be addressed by using mPDR-HS, where the pre-launch route search process finds four individually feasible segments, namely, $N_{\text{1}}\to N_{\text{3}},N_{\text{3}}\to N_{\text{5}},N_{\text{5}}\to N_{\text{6}}$ and $N_{\text{6}}\to D$. As shown, the launched packet header at source $N_{\text{1}}$contains the tuple {launching speed, launching angle, gravitational direction for mPDR computation at the intermediate nodes, and the segment end point} for all those four segments. For example, the information about the first segment contains $N_{\text{3}}$ as segment end point, launching speed $s_{\text{1}}$, angle $\theta_{\text{1}}$, and the field direction to be used for mPDR computation by all the intermediate nodes in this segment. Subsequently, node $N_{\text{3}}$ uses the information from the packet header about the next segment to launch the packet towards the correct next hop, and the packet eventually reaches the segment end-point $N_{\text{5}}$. This process is followed at all subsequent segments till the packet reaches destination node D.

## 8 PDR route quality and multicast

### 8.1 Minimum-hop routes

For a given field distribution, the PDR route of a packet within a discretized topology space can be non-linear. For example, under downward gravity, a packet’s route will be an approximated parabolic projectile. Unlike with classical routing, such non-linearity may prevent a PDR route from being minimum-hop.

One special case to straighten a projectile-style trajectory is to use a very high launching speed directly towards the destination point. However, this does not usually work with PDR because of space discretization in a topology, which prevents the packet to be launched in a directly targeted way towards the destination node.

**Fig 5:**
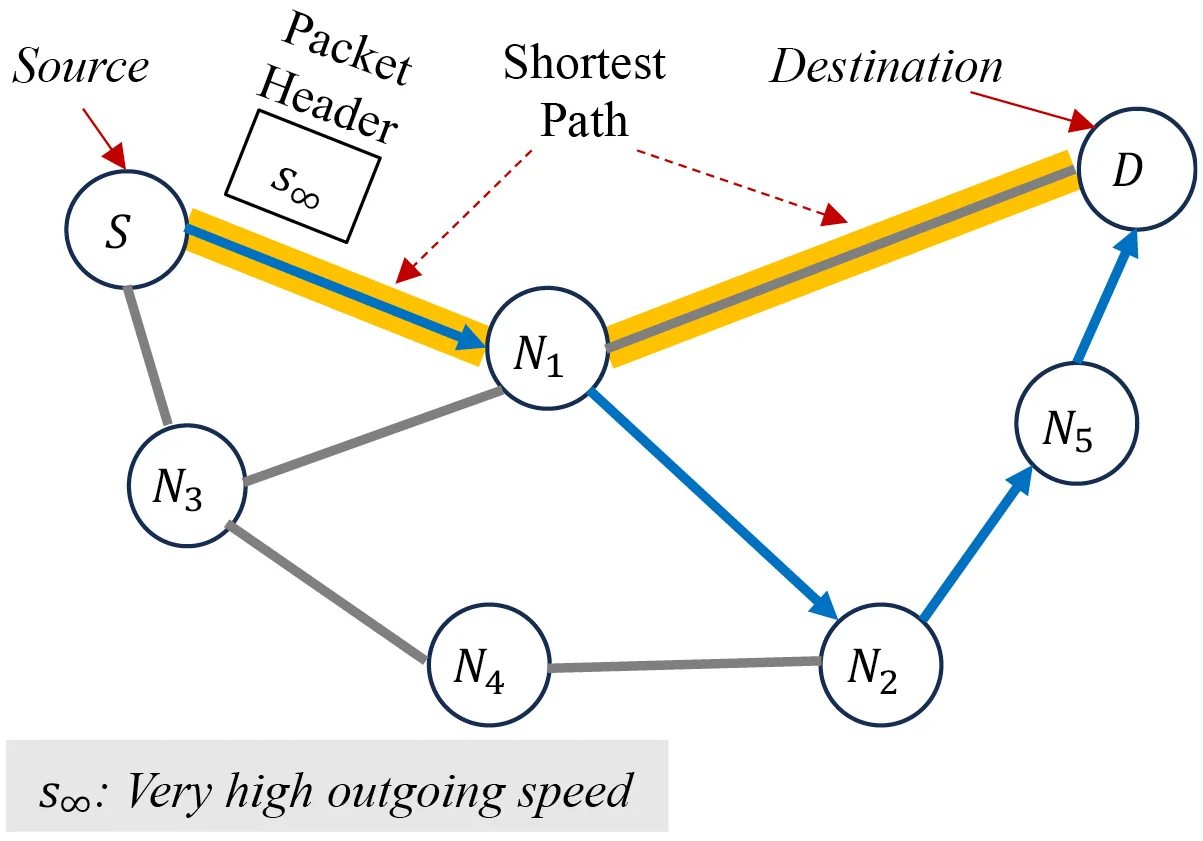
PDR case with very high launching speed.

An example scenario is depicted in Fig 5 in which a packet is launched from source node S to a destination node D with an arbitrarily high launching speed. Now, consider if the chosen launching angle corresponds to $NodeN_{\text{1}}$, which is along the minimum-hop route. The PDR execution at $N_{\text{1}}$ results in $N_{\text{2}}$ as the next hop, and execution at $N_{\text{2}}$ results $N_{\text{5}}$ as the next hop. So, the packet finally reaches destination D via the route $S\to N_{\text{1}}\to N_{\text{2}}\to N_{\text{5}}\to D.$ This example demonstrates that minimum-hop PDR may not be feasible even when a packet is launched with a very high speed, and with an angle that corresponds to one of S’s outgoing links that points as close to the physical direction of D as possible.

### 8.2 Multicast PDR

In multicast routing scenarios, PDR handles packet delivery by designing trajectories that sequentially traverse multiple destination nodes belonging to a multicast group. This is achieved during the pre-launch route search phase, where candidate trajectories are evaluated not only for reachability to a single destination but also for their ability to intersect multiple intended receiver nodes along a feasible path. Once such a trajectory is identified, packet forwarding proceeds using the same local trajectory computation principles as in unicast routing [32], without requiring route replication at intermediate nodes. This trajectory-centric multicast realization allows a single packet instance to serve multiple destinations along its path, although it may introduce trade-offs in terms of route optimality and increased search complexity when the multicast group size grows.

In contrast, the baseline PDR and its enhancements target one-to-one unicast trajectories. The multicast version of PDR, as mentioned before, can be achieved by pre-launch searches for trajectories that passes through a set of destination nodes which belong to a multicast group. The only difference here is that the list of destination node addresses or a relevant multicast group address should now be added to the packet header. This would enable the packet to be PDR-routed till the last node in the corresponding multicast group receives the packet. From a performance standpoint, since the PDR trajectories are inherently non-minimum-hop, converting a unicast PDR route to a multicast one may not add significant number of additional hops to a route. As stated before, the pre-launch route search complexity, however, would be higher with larger number of members in the destination multicast group.

## 9 Privacy semantics of PDR

In PDR, the intermediate nodes do not require to know the identity of a packet’s destination. This allows the destination address in the packet header to be encrypted with either a source-destination shared key, or with the public key of the destination. Similarly, in mPDR with Hierarchical Source Routing (mPDR-HS), all the segment end-points are encrypted with the keys related to the respective end points. With such an encryption arrangement, for all versions of PDR, just by looking at a packet, an eavesdropping entity cannot know about the identity of the destination node. Same applies for the multicast version of PDR.

This privacy protection, however, weakens as an adversarial entity possesses more information about nodes’ true coordinates in space. Consider a situation when an adversarial eavesdropper is listening to a packet over the wireless link $N_{\text{1}}\to N_{\text{2}}$. It can have access to the following unencrypted information from the packet: i) outgoing speed from $NodeN_{\text{1}}$, and ii) gravity direction to be used for PDR by $NodeN_{\text{2}}$. Now, Eqns. 8–17 indicates that if somehow the adversarial agent gets access to the coordinate of $NodeN_{\text{1}}$, it can compute the coordinate of $NodeN_{\text{2}}$. This way, the identity of more downstream nodes can be iteratively compromised as the agent gets the physical coordinate information about more nodes in the topology. It also turns out that in the presence of node coordinate information, the identity of upstream nodes in a trajectory, including the source, can be extracted by executing the PDR equations in the reverse order. This requires that in PDR framework, in addition to destination identity encryption, care must be taken to conceal the node coordinates.

### 9.1 Potential trajectory inference threat by adversarial nodes

Despite the inherent privacy advantages of avoiding explicit destination disclosure in packet headers, PDR may still be susceptible to trajectory inference attempts by adversarial intermediate nodes. In particular, if an adversarial entity has access to accurate network topology information and knowledge of the logical field distributions, it may attempt to estimate the likely continuation of a packet’s trajectory based on observed incoming link direction and header-encoded motion parameters. Such inference, however, typically remains probabilistic due to discretization effects, trajectory approximation at intermediate nodes, and the possibility of multiple feasible downstream paths under varying field configurations. Consequently, while PDR reduces direct exposure of destination identity, additional security mechanisms such as trajectory randomization, dynamic field adaptation, or route segmentation may be explored in future extensions to further mitigate inference risks.

## 10 Experimental setup and results

### 10.1 Pre-launch PDR route search algorithm

Before a packet is launched, an algorithmic search is executed for finding; a) a feasible trajectory, and b) a launch speed, launch angle, and gravity direction (i.e., for mPDR) in order to realize the trajectory. This functionality can be achieved either by using a routing algorithm, or by a heuristics-based search process. While such algorithms are candidates for future research, for the experiments in this paper we have used a heuristics-based search process as outline below. Note that this search entity can reside on the source node itself, or on a separate route compute server.

The search algorithm’s inputs consist of the network topology, the source and destination nodes, and an assigned gravity vector at all nodes. The algorithm iterates over all the links from the source node to identify feasible trajectories that pass through the destination node or nodes in case of multicast. While iterating on a link, the algorithm first determines a speed beyond which the trajectory remains unchanged. This is accomplished using an exponential search [33], where in each step the speed is increased by powers of two until the resulting trajectory does not change. Once such an upper bound of speed is established, a binary search [34] is conducted within the range specified by an arbitrarily small value and the upper bound. This process finds all unique trajectories through the link (i.e., being iterated on) corresponding to different speed values. In other words, at the conclusion of the binary search for a link of the source node, all possible trajectories through that link is enumerated. Consequently, after the conclusion of the search for all links of the source node, the complete set of possible network-wide trajectories from the source, and the corresponding (outgoing link, speed) tuples for those trajectories are known. The quality of the trajectories in terms of hop-count or any other metric can also be recorded during the search process. Note that the search process described so far assumes a specific gravity vector across all the network nodes. In case of m-PDR the search process is repeated for all the allowed gravity vectors as explained in Section 7.

The final step involves selecting a trajectory from the complete set of possible trajectories based on the desired quality requirements or multicast objectives. After a packet is launched on the selected trajectory based on the corresponds outgoing link and launching speed, all the downstream nodes in the network executes the PDR algorithm presented in the prior sections.

### 10.2 Results and analysis

#### 10.2.1 Trajectories with different launching speeds and gravity vectors.

**Fig 6:**
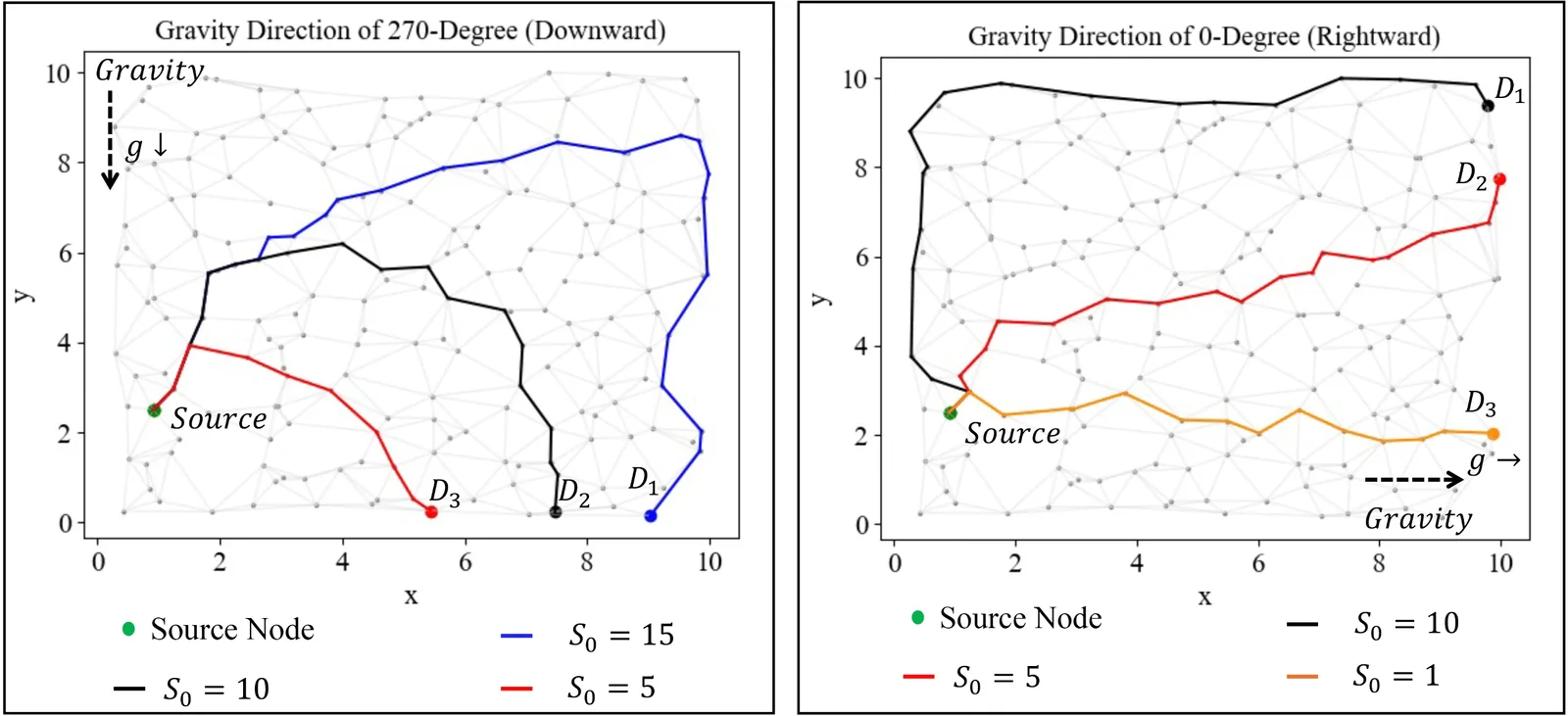
Impacts of launching speed and gravity directions on *PDR* trajectory.

Experiments were carried out for observing PDR trajectories on a topology of 200 nodes. Fig 6 depicts the trajectories resulting from packets being launched from a source node with different speeds, but through the same outgoing link. The network topology and trajectories are shown in a 2-dimensional coordinate space.

For the trajectories in Fig 6(a) and 6(b), the gravity vector points downwards and rightwards respectively. Following observation can be made. First, the PDR trajectories on the discrete topology space somewhat resemble the parabolic trajectory of continuous space projectiles. The diversion from the continuous-space parabolic trajectory can be explained by the topology-induced discrete-space constraints as introduced in Section 3. The second observation is that by varying the launching speed, the PDR trajectories from a source can be modulated for reaching different destinations. This is evident from Fig 6(a) where a high launching speed (i.e., $s_{\text{0}}=\text{15}$) resulted in a packet following the blue trajectory, leading to the topology-edge node $D_{\text{1}}$. By reducing the launching speed, the trajectories can be modified for reaching other topology-edge nodes such as $D_{\text{2}}$ and $D_{\text{3}}$. Similar observations can be also made in Fig 6(b). The final observations from Fig 6 is that different gravity directions can be effectively used for mPDR trajectory control. For instance, in Fig 6(a) and 6(b), trajectories under downward and rightward gravity directions differ significantly even for the same packet launching speeds $S_{\text{0}}=\text{5}$ and 10. These observations are found to be generalized via validations other mesh topologies and trajectories.

#### 10.2.2 Effects of gravity directions on reachability.

**Fig 7:**
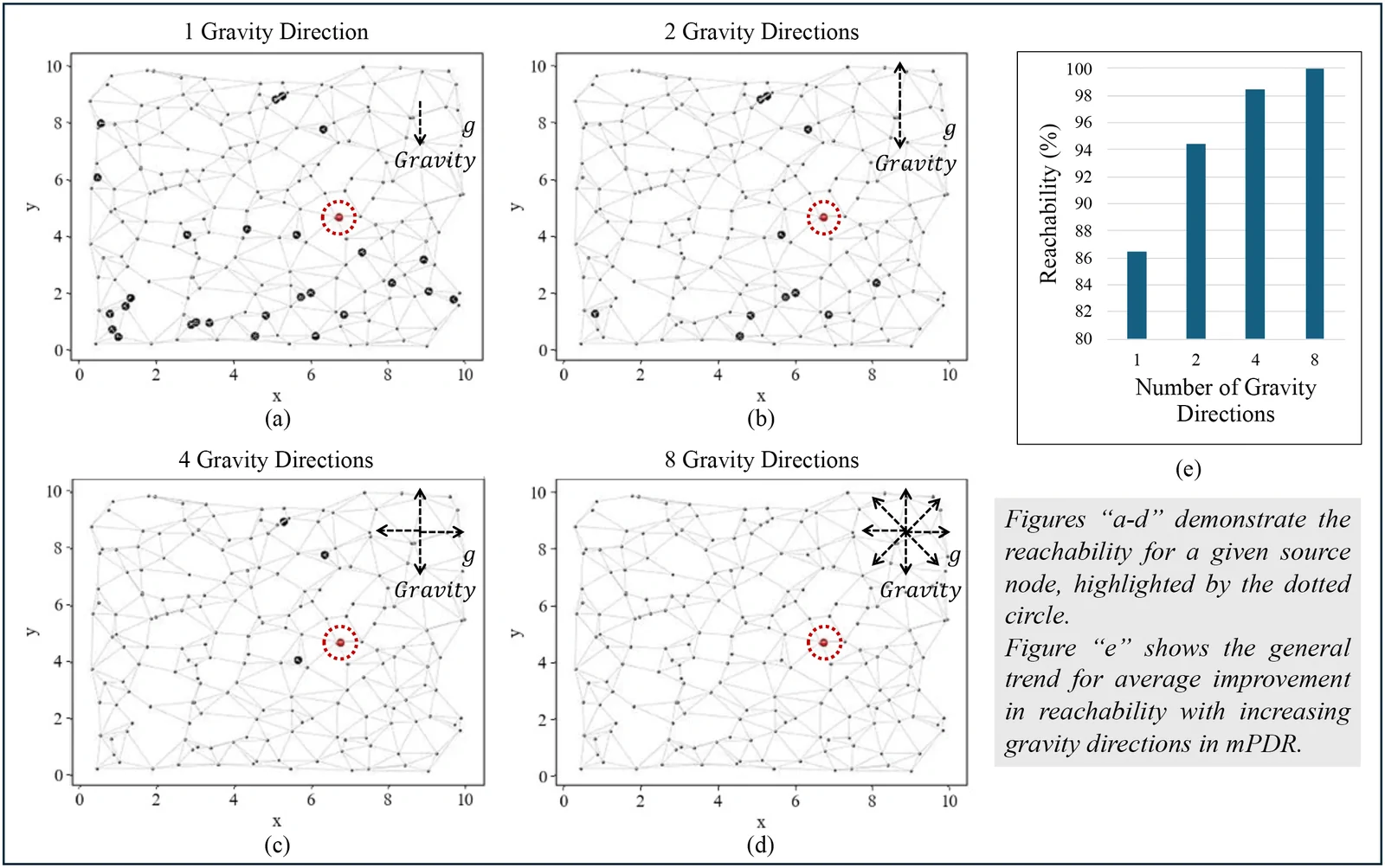
Effects of the number of gravity directions on *mPDR* reachability.

Experiments were performed for understanding PDR’s reachability, and how it can be improved using multi-field PDR (mPDR) as presented in Section 7. In the same topology shown in Fig 6, when the gravity vector points downwards, Fig 7(a) depicts all unreachable nodes (shown in black) from a given source node (shown in red). Out of 199 possible destinations, 27 are not reachable in this case. Fig 7(b) shows that for mPDR with two gravity directions (i.e., up and down), the number of unreachable nodes goes down to 11. Similarly, as shown in Figs. (c) and (d), when mPDR is allowed to use 4 and 8 different gravity directions, the number of unreachable nodes reduces to 3 and 0 respectively. These results demonstrate that although not guaranteed, complete or near-complete reachability can be achieved by allowing mPDR routing to use a large number of diverse field directions.

**Fig 8:**
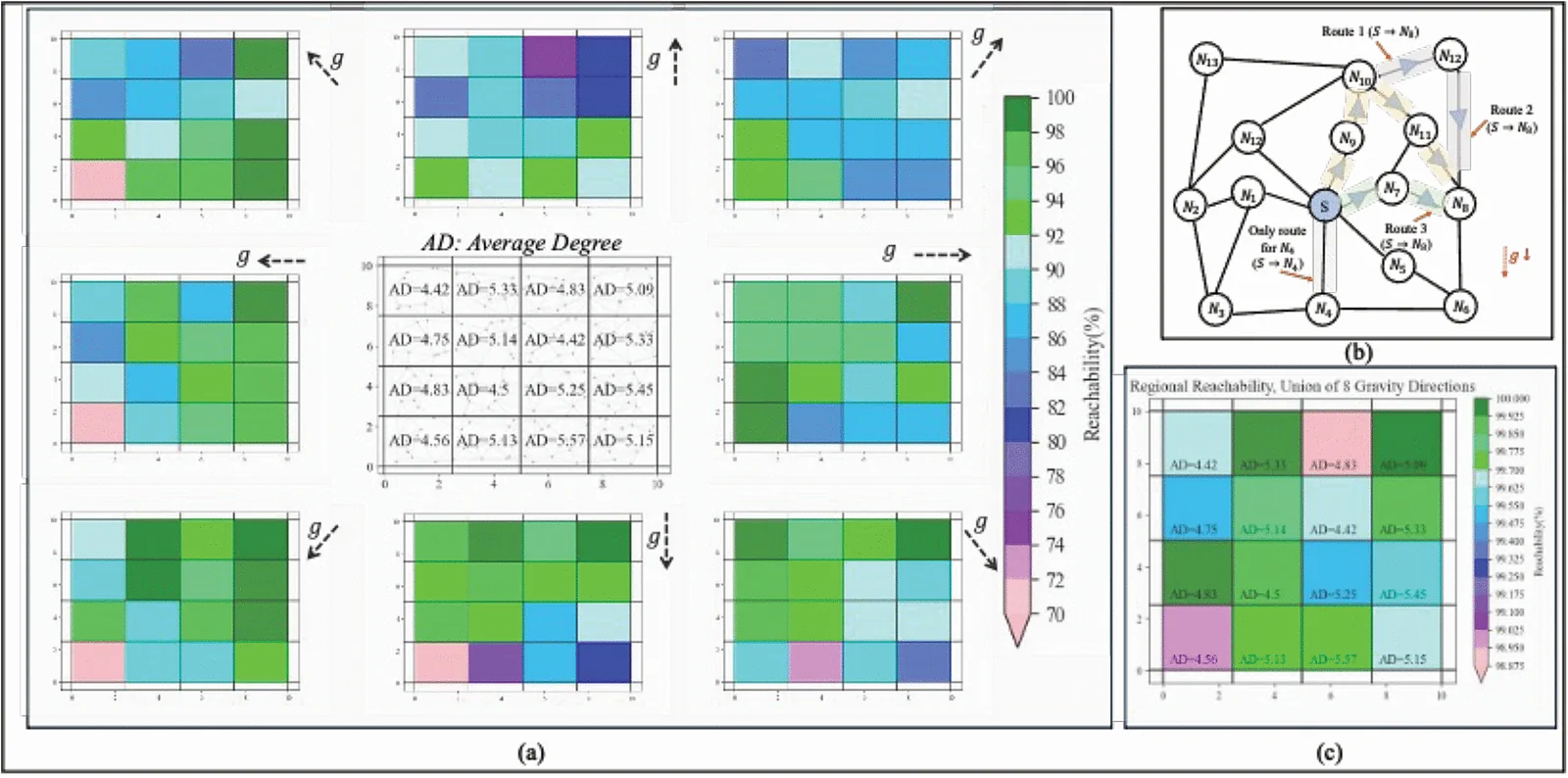
Regional reachability map with various gravity directions.

Region by region mPDR reachability for different gravity directions is demonstrated in Fig 8(a). These results are shown for the same 200-nodes topology in Fig 6. The topology terrain is divided into 16 distinct 2.5×2.5 sq. unit regions. Average mPDR reachability to destinations in each of those regions from the rest of the network is reported. It can be observed from Fig 8(a) that for a given gravitational field direction, the average reachability to the region that is situated in the direction of the field is lower than those in regions that are in other directions. For example, when gravity is downwards, the reachability is lower for the regions at the bottom of the topology.

Note that the central part of Fig 8(a) presents a grid-based network topology analysis where AD denotes the average degree of connectivity within each specific grid cell. This representation aids in assessing spatial reachability, thus indicating how well each region within the network topology facilitates communication among nodes.

This somewhat counterintuitive effect can be explained by considering a simple topology shown in Fig 8(b) where node S is the source, and the gravity is pointing downwards. In this case, consider destination node $N_{\text{8}}$ which is located rightward with respect to the source S. For this node, there exists multiple possible routes from source S to reach it. The same logic applies for node $N_{\text{2}}$ located towards the left of S and so on. On the other hand, for node $N_{\text{4}}$, which is located in a direction along the downward gravity, the only way to reach it from node S is to launch the packet directly downwards (along link $S\to N_{\text{4}}$). In the absence of a link, node $N_{\text{4}}$ becomes non reachable form S.

Fig 8(c) shows the effective reachability after taking the union of reachabilities for all eight gravity directions, as done in mPDR. It shows that mPDR with 8 different field directions can deliver average reachabilities of more than 98% in all the regions. This result further reinforces the effectiveness of the mPDR protocol as presented in Section 7.

#### 10.2.3 Effects of network degree on reachability.

**Fig 9:**
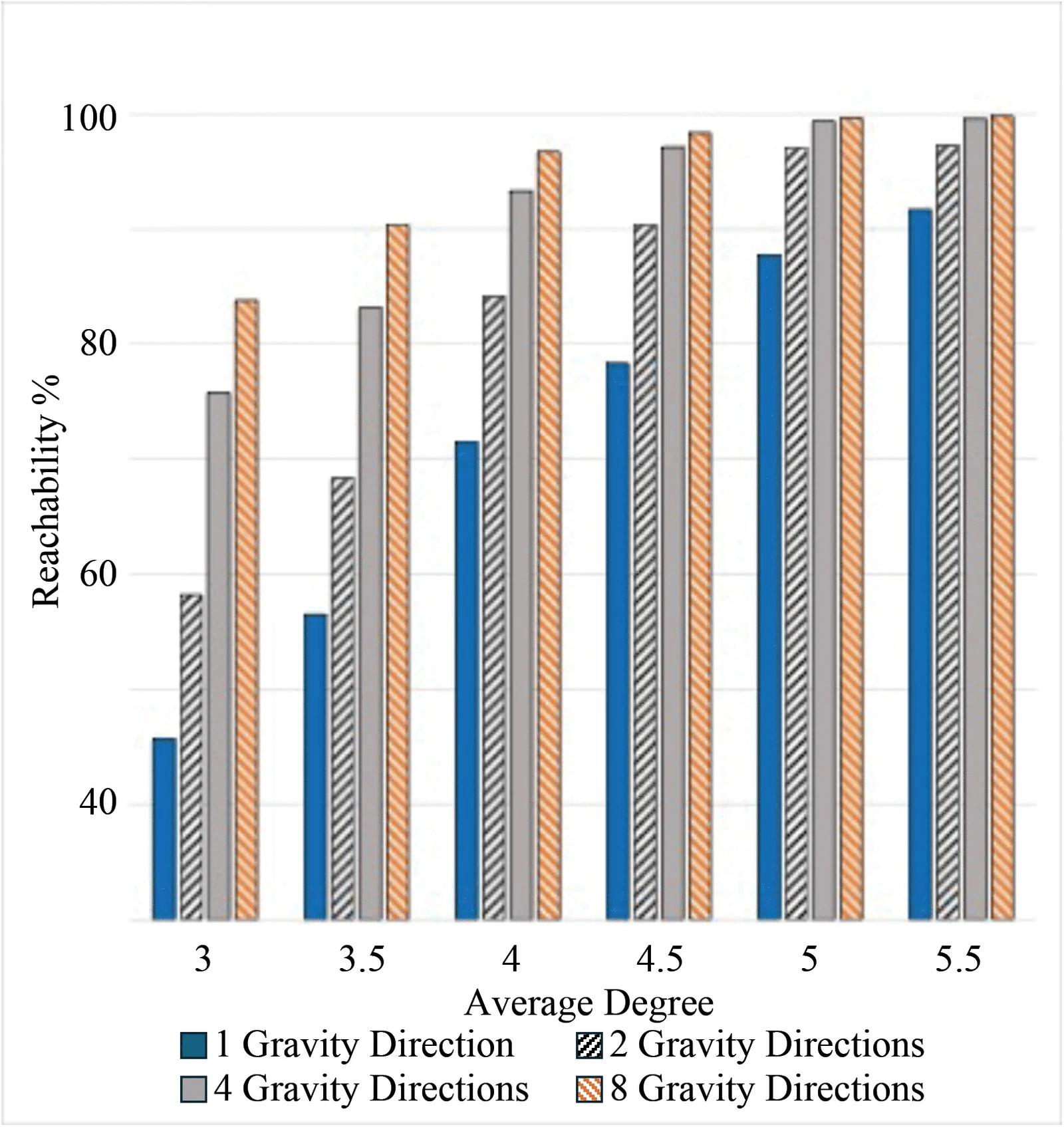
Effect of network degree and number of gravity directions on *mPDR* reachability.

Fig 9 explains the effects of network degree and gravity vector diversity with multi-field PDR (mPDR) on reachability. The experiments, carried on 200-node networks, show a clear correlation between network degree and packet reachability. With increasing network connectivity degree, a topology becomes less discretized and move towards the continuous space projectile scenarios. This reduces the artifacts associated with field discretization, and enhances the likelihood of finding viable routing paths, thereby improving reachability.

The figure also shows how the reachability can be further improved significantly by incorporating more gravity directions. For example, allowing 8 distinct field vectors in networks with an average degree of 5.5 elevates reachability to nearly 99.75%. Interestingly, while the addition of multiple gravity vectors generally promotes reachability, its impact diminishes in denser networks. In dense networks, often a solitary gravity vector (i.e., mPDR with m set to 1) proves sufficient for sufficient reachability. This indicates that the complexity introduced by additional vectors may not be justifiable in strongly connected networks.

**Fig 10:**
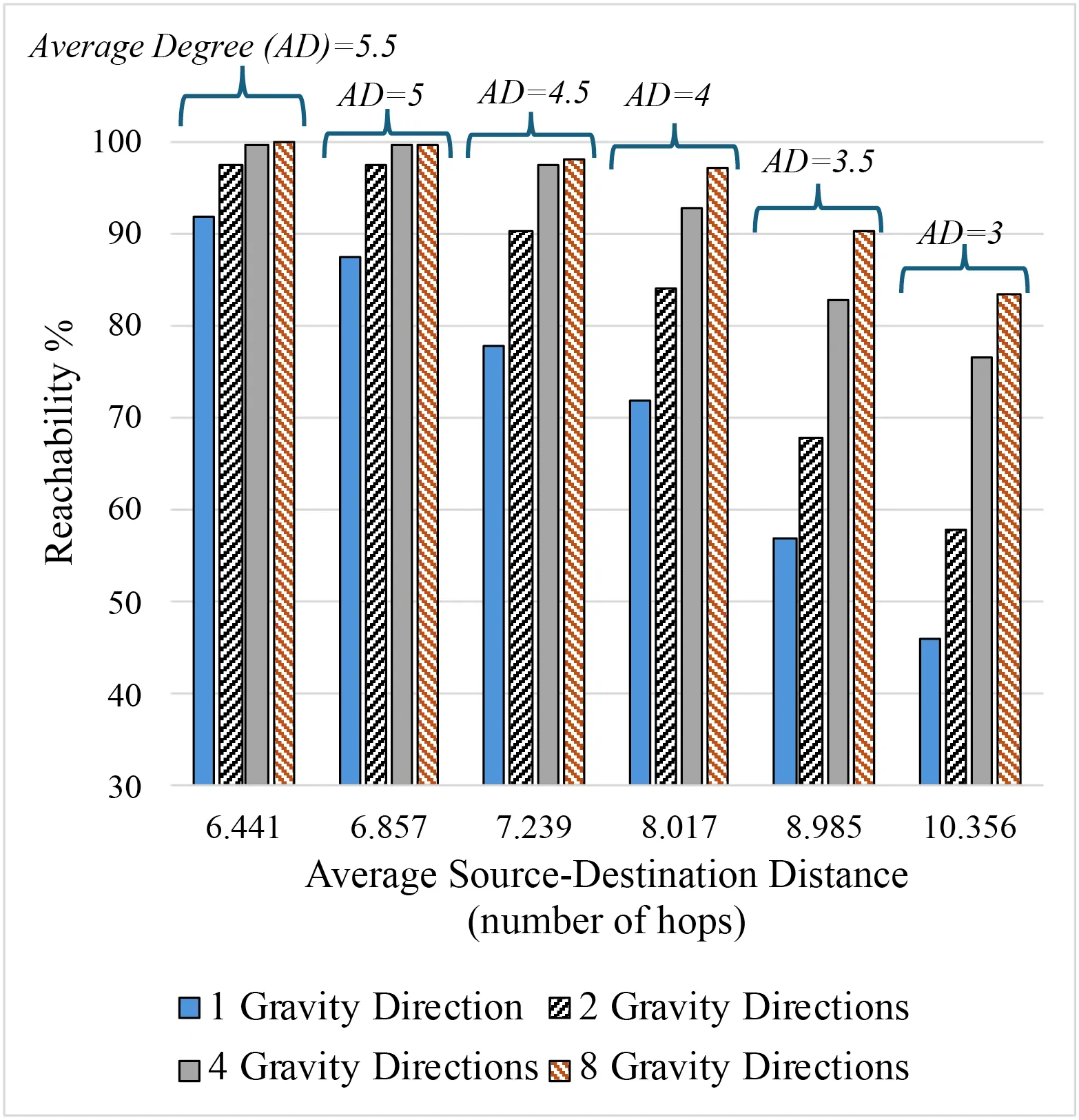
Effects of shortest path length on *mPDR* reachability.

Fig 10 shows the effects of network degree on PDR’s reachability via the average route length. Average shortest path length (in hops) in a network generally reduces with higher network degree. As shown in the figure, with various topologies in which the average node degree is varied between 3 and 5.5, the resulting shortest path length changes between 6–11 hops, approximately. The figure clearly shows, as the average shortest path length reduces (i.e., higher node degree), the PDR reachability increases for the same reasons found for the results in Fig 9. The impacts of number of gravity directions in Fig 10 are as expected. That is with higher number of gravity vector directions, mPDR demonstrates better overall network-wide reachability.

#### 10.2.4 Route quality with particle dynamics based routing.

**Fig 11:**
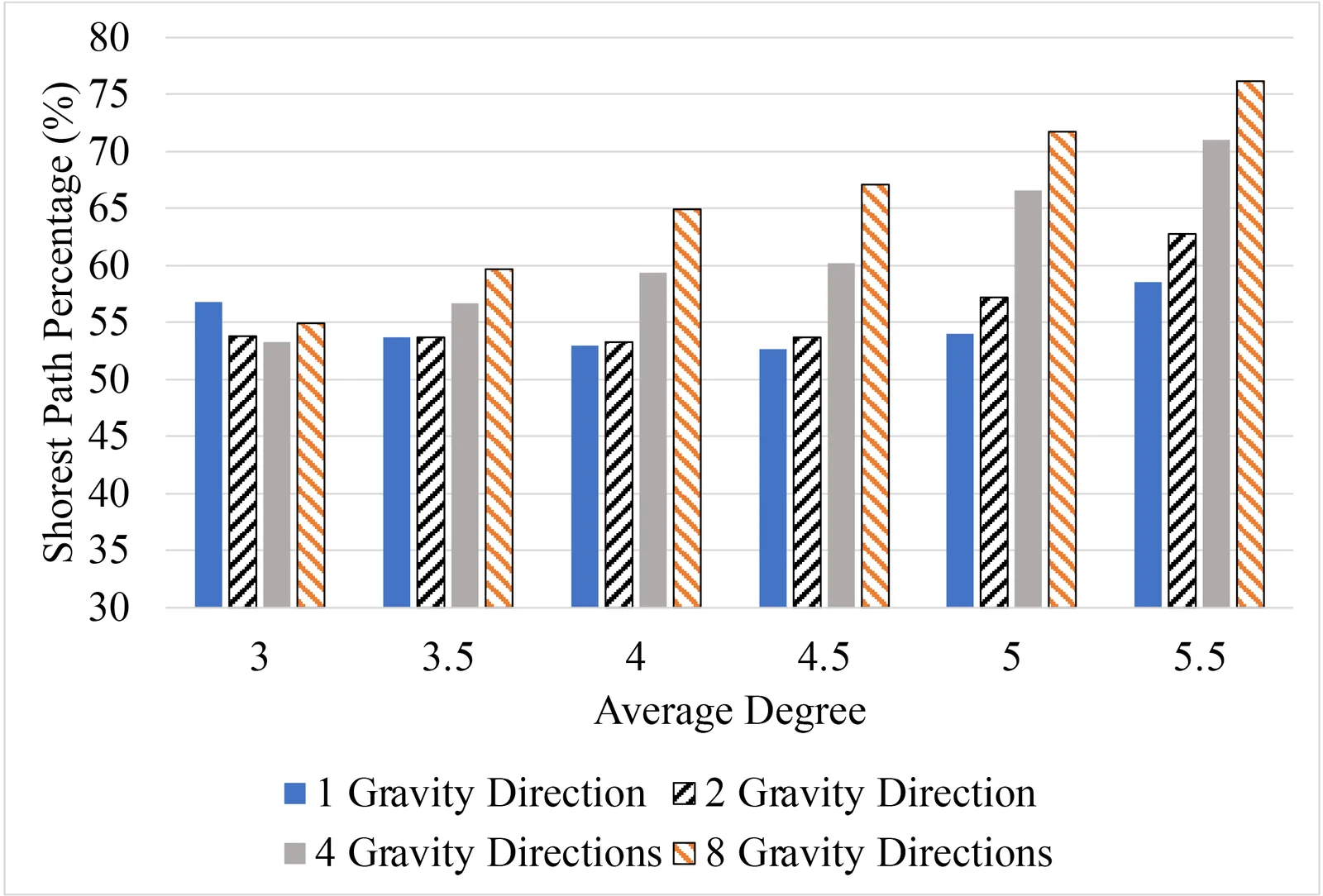
Quality of PDR routes with network degrees and gravity directions.

One notable quality tradeoff of particle dynamics-based routing is its inability to deliver end-to-end shortest paths, which can be guaranteed by classical routing algorithms. Fig 11 illustrates PDR path quality expressed in terms of the percentage of all experimented PDR paths that are the shortest according to Dijkstra’s shortest path algorithm [10]. The figures shows a general trend where the percentage of shortest paths increases with network degree, indicating better PDR/mPDR path quality for denser networks. This trend is especially pronounced for mPDR with multiple available gravity directions. This demonstrates mPDR’s ability to exploit additional gravitational fields for optimizing routing paths, especially for denser networks. In summary, unlike classical routing, even though the approach cannot absolutely guarantee shortest path routing, for dense networks, the mPDR algorithm can ensure a high percentage of routes to be the shortest.

## 11 Summary and conclusions

This paper introduced a novel packet routing mechanism inspired by the principles of particle dynamics in force fields. Unlike traditional hop-by-hop routing, the proposed Particle Dynamics-based Routing (PDR) eliminates the need for forming routing tables and table-based forwarding. Instead, it relies on force field-based trajectory computation equations for packet forwarding. Packets in PDR require only two constant-size header fields, namely, speed and force field vector direction. Being agnostic to route length, this provides a distinct scalability advantage over source routing, where packet header length increases with network size. With random mesh topologies of up to 200 nodes and gravity as an example force field, our experiments show that PDR trajectories do resemble approximated (i.e., discretized) projectiles, which can be successfully used for packet routing. The experiments further showed that adjusting launching velocity and gravity vector directions, PDR can modulate trajectories to reach different destinations efficiently. We have also explored the impacts of gravity directions on PDR reachability and proposed a multi-field PDR (mPDR) approach to enhance reachability by leveraging diverse field directions. In summary, by leveraging generalized in-field particle dynamics principles, the PDR framework was shown to offer promising routing solutions that can move complexity from network core to route search/computation entities at the edge.

Future works on this research will include extending the concept to other force fields such as electrostatic, electromagnetic fields etc. Another important direction would be to devise routing mechanism for non-planer topologies, which will involve particle trajectories under force fields in 3-dimensions. Furthermore, this will require exploration of multi-objective evolutionary solutions for possible NP-hard problem encountered while applying logical field in a 3-dimensional topology [35,36]. Finally, algorithmic route computation mechanisms will need to be devised for complementing the route search mechanisms used in this work.
